# Giant Mesenteric Lymphangioma in a Young Adult: A Rare Clinical Entity

**DOI:** 10.1155/cris/6779801

**Published:** 2026-05-03

**Authors:** Abdo Imad El Tawil, Leandro Alencar Furtado Machoski, Beatriz Arnaut Mendes, Micheli Fortunato Domingos, João Carlos Chiquetto Filho, Fernando Issamu Tabushi, Eduardo José Brommelstroet Ramos

**Affiliations:** ^1^ Department of Medical Research, Faculdade Evangélica Mackenzie do Paraná, R. Padre Anchieta 2770 - Bigorrilho, Curitiba, 80730-000, Paraná, Brazil; ^2^ Department of General and Digestive Surgery, Hospital Nossa Senhora das Graças, R. Alcides Munhoz 433 - Mercês, Curitiba, 80810-040, Paraná, Brazil; ^3^ Department of Human Anatomy, Universidade Federal do Paraná, R. XV de Novembro 1299 - Centro, Curitiba, 80060-000, Paraná, Brazil, ufpr.br

**Keywords:** gastrointestinal diseases, lymphangioma, mesenteric cyst, surgical approach

## Abstract

Cystic lymphangioma is a rare benign tumor of lymphatic origin, characterized by slow growth and variable clinical presentation. The presence of a peritoneal diverticulum is exceedingly rare in adults and often poses a diagnostic challenge owing to nonspecific symptoms and imaging findings. A 22‐year‐old previously healthy male presented with abdominal fullness and flatulence without other relevant symptoms. On physical examination, a firm, palpable abdominal mass was identified. Laboratory tests revealed mild elevations in alanine aminotransferase and alkaline phosphatase levels. Abdominal computed tomography (CT) revealed a cystic lesion in the epigastric and right hypochondriac regions adjacent to the liver, pancreas, and stomach. Magnetic resonance imaging (MRI) revealed a cystic mass suggestive of lymphangioma, measuring 21 cm × 20 cm × 16 cm. The patient underwent open retroperitoneal lymphadenectomy with complete excision of the lesion and additional foci located above the superior mesenteric vein. Histopathological examination confirmed the diagnosis of mesenteric cystic lymphangioma. Mesenteric lymphangiomas are rare in adults and present with vague, nonspecific abdominal symptoms. Imaging studies play an essential role in diagnosis, although definitive confirmation requires histopathological evaluation of the tissue. Complete surgical resection remains the treatment of choice and is critical for preventing recurrence and potential complications, such as infection, rupture, or intestinal obstruction. Given the rarity and diagnostic challenges of mesenteric cystic lymphangioma in adults, clinicians should maintain a high index of suspicion when evaluating abdominal cystic lesions in adults. Early surgical intervention is fundamental for achieving optimal outcomes and preventing future complications.

## 1. Introduction

Lymphangiomas are rare benign tumors of lymphatic origin, with a higher prevalence in the cervical (75%) and axillary (20%) regions, whereas their occurrence within the abdominal cavity is considered uncommon (5%) [1, 2]. Among abdominal lymphangiomas, mesenteric localization represents only a small proportion of reported cases, accounting for less than 1% of all lymphangiomas [2, 3]. Since their first description by Benevienae in 1507, these lesions have remained exceedingly rare, with modern estimates suggesting an incidence of ~0.6 per 100,000 surgical pathology specimens [2–4]. Adult and pediatric intra‐abdominal multicystic lymphangiomas collectively represent fewer than 1% of all lymphatic malformations [4]. The estimated incidence of lymphangiomas in the peritoneal cavity is ~1 in 100,000 adults and 1 in 20,000 children [2].

The pathogenesis of mesenteric lymphangiomas remains controversial; however, the most widely accepted hypothesis suggests a congenital malformation of the lymphatic system resulting from developmental failure or acquired obstruction of lymphatic vessels, leading to cystic formation [1, 5]. These lesions are often asymptomatic and incidentally diagnosed; however, they may also present with abdominal pain, distension, a palpable mass, or, in rare cases, an acute abdomen due to complications such as volvulus, hemorrhage, or rupture [6, 7].

Lymphangiomas are histologically characterized by dilated cystic or vascular spaces lined by a single layer of flattened endothelial cells within a fibrocollagenous stroma, frequently containing lymphocytes [8]. In mesenteric locations, they demonstrate dilated and proliferative lymphatic vessels with thin, well‐demarcated walls and no intercommunication [9]. Although benign, they have the potential for local invasion [9, 10]. The differential diagnoses include hemangiomas, branchial cysts, lipomas, cystic mesotheliomas, rhabdomyosarcomas, and parasitic diseases such as hydatidosis [11–13].

Diagnostic imaging plays a crucial role in the characterization of these lesions, with computed tomography (CT) and magnetic resonance imaging (MRI) being the main modalities used to evaluate mesenteric lymphangiomas [7, 11, 14, 15]. Nevertheless, a definitive diagnosis can only be established through histopathological examination [7]. Complete surgical excision remains the treatment of choice, as incomplete resection may lead to recurrence [11, 16, 17].

Despite its rarity, mesenteric lymphangioma should be considered in the differential diagnosis of cystic abdominal masses, particularly in pediatric and young adult patients, given its potential for serious complications, such as torsion, hemorrhage, infection, and rupture [4, 12, 18].

Herein, we present the case of a 22‐year‐old male with no prior medical history other than obesity, who presented with nonspecific abdominal symptoms and was found to have a giant mesenteric lymphangioma, which was successfully treated with complete surgical excision.

## 2. Case Presentation

A 22‐year‐old previously healthy man with no relevant medical history presented with recurrent episodes of abdominal fullness and flatulence that started 5 months before. His body mass index was 31.6 kg/m^2^. The patient denied experiencing abdominal pain, nausea, vomiting, bowel habit changes, fever, or other systemic symptoms. No additional lymphangiomatous anomalies were identified, and there was no family history of lymphatic diseases. The patient denied recent use of any medications. On physical examination, he appeared to be in good general condition, hemodynamically stable, afebrile, and anicteric. The abdomen was soft and nontender to both superficial and deep palpation, with no signs of peritoneal irritation or visceromegaly, but presented a large palpable mass in the mesogastrium and epigastrium. Laboratory tests revealed mild hepatic enzyme elevations, with elevated alanine aminotransferase at 69.2 U/L, gamma‐glutamyl transferase at 51.9 U/L, and alkaline phosphatase at 298 U/L, while other hematologic, biochemical, and inflammatory parameters were within the normal range. Tumor markers, such as carcinoembryonic antigen and CA19‐9, were also within the normal range.

**Figure 1:**
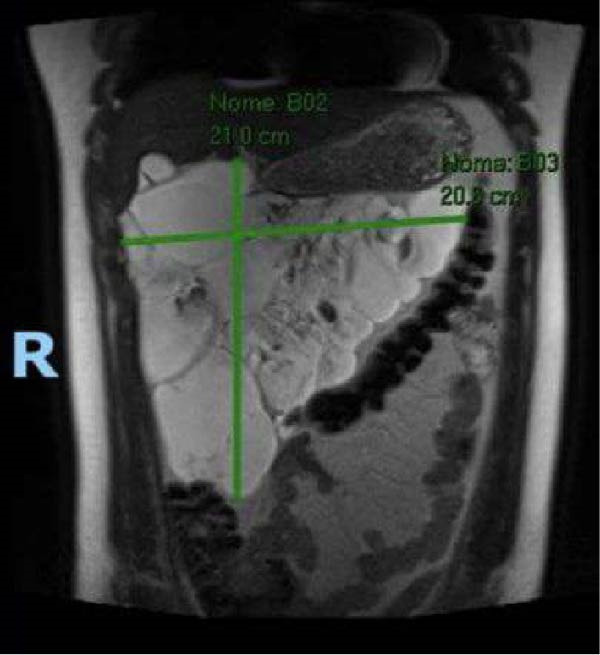
Coronal T2‐weighted MRI demonstrating a multiloculated cystic lesion measuring 21.0 cm × 20.8 cm within the abdominal cavity, characterized by thin internal septations, a thin and smooth wall, and homogeneous hyperintense fluid content. The lesion exerted a significant mass effect on the adjacent bowel loops and liver. The imaging findings were consistent with cystic lymphangioma.

Based on the laboratory findings and the patient’s clinical picture, further imaging studies were performed. Abdominal CT revealed a large cystic lesion in the epigastric region and right hypochondrium. The lesion had an unspecified origin and extensively involved the gastric body and antrum, as well as the duodenal arch, and was in close contact with adjacent structures, including the right hepatic lobe, pancreatic head, and transverse colon. It preserved a well‐defined cleavage plane with the inferior vena cava and portal vein, with no evidence of vascular invasion or significant compression of the major vessels. For better characterization, abdominal MRI was performed, confirming a voluminous multiloculated cystic lesion measuring 21 cm × 20 cm × 16 cm (Figure 1), which extended from the right flank through the right hypochondrium and epigastric regions. The lesion exhibited no infiltration of adjacent organs but maintained intimate anatomical relationships with the surrounding structures.

**Figure 2:**
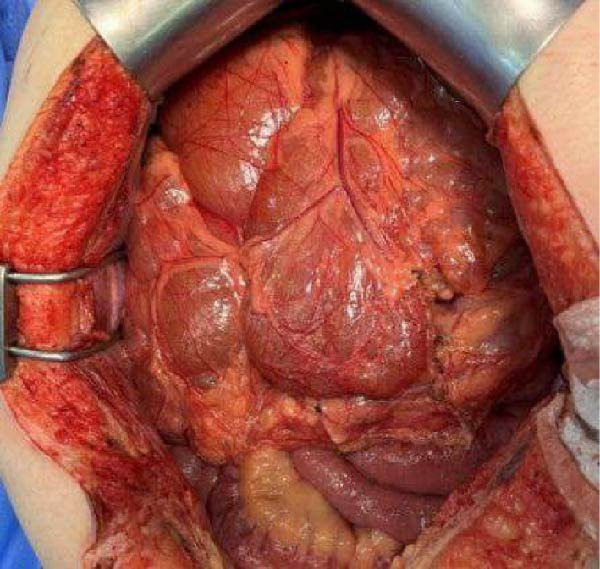
Intraoperative view: macroscopic in situ appearance of a multilobulated, well vascularized mesenteric mass during surgical excision.

Considering the suspected diagnosis and the large size of the lesion, which posed a risk of progressive compression of the abdominal organs, surgical management was indicated. No previous biopsy or fine‐needle aspiration (FNA) was performed because of the good radiological characterization of the lesion and the lack of evidence of benefit from these procedures before surgical removal. Five months after the primary investigation, the patient underwent open laparotomy for complete resection of the lesions (Figure 2). During the procedure, an additional focus of disease located above the superior mesenteric vein was excised along with the affected lymph nodes in the mesenteric root and mesocolon. Significant biliary compression by the tumor was observed intraoperatively, which could explain the elevation of hepatic enzymes observed during the initial investigation. The total operative time was 6 h and 55 min, with no intraoperative complications.

**Figure 3:**
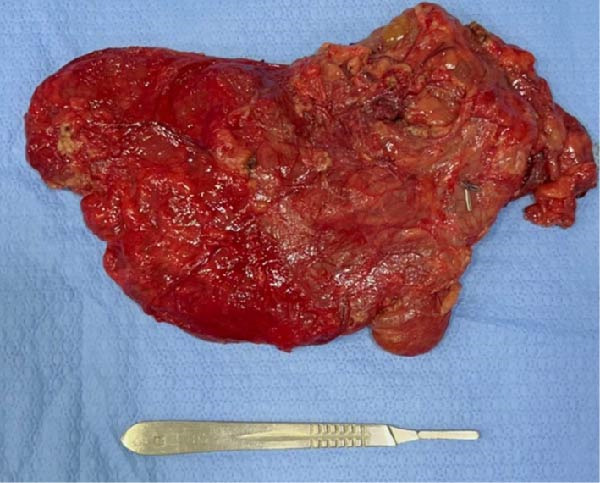
Gross specimen demonstrating a large, multiloculated cystic lesion.

**Figure 4:**
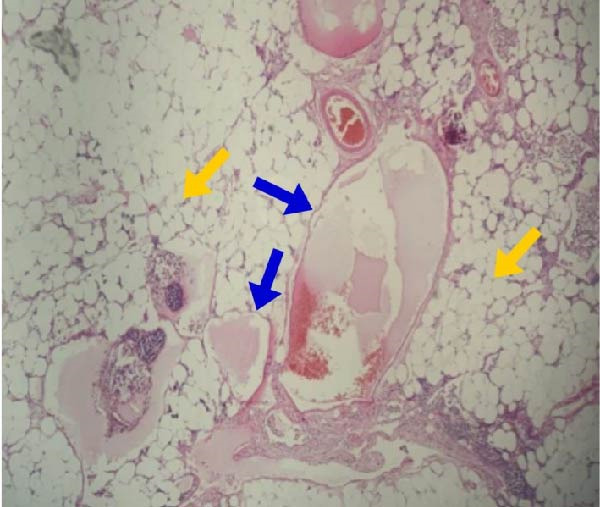
Histopathological analysis of the removed specimen (40x H&E) showing dilated lymphatic channels lined by a single layer of nonatypical endothelial cells (blue arrows) within mature adipose tissue (yellow arrows), confirming the diagnosis of cystic lymphangioma.

The resected specimen (Figure 3) was sent for histopathological examination, which confirmed the diagnosis of mesenteric lymphangioma, with no evidence of malignancy or lymph node involvement by other pathologies (Figure 4). The total hospital stay was 5 days, including 2 days in the intensive care unit. Postoperatively, the patient had an uneventful recovery, with no surgical complications or recurrence of the lesion to date.

During a 12‐month follow‐up period, laboratory tests demonstrated normalization of hepatic enzyme levels within the institutional reference ranges. Radiological evaluation with MRI revealed no evidence of residual or recurrent cystic lesions. The patient remained clinically asymptomatic, confirming complete clinical, biochemical, and radiological remission after surgical treatment. No late complications of the surgery were reported.

## 3. Discussion

Lymphangiomas in the peritoneal cavity are extremely rare in adults [1, 2]. Although they may occur in the retroperitoneum and omentum, the mesentery, owing to its rich lymphatic network, is also a potential abdominal site of involvement [2, 3]. The clinical presentation of abdominal cystic lymphangiomas is variable and nonspecific, often providing little information for establishing a clinical diagnosis [5].

In the present case, the patient exhibited vague symptoms such as abdominal fullness and flatulence, with no clinical signs suggesting a large abdominal lesion. Physical examination was remarkable only for a huge mass palpated in the abdomen, emphasizing the diagnostic challenge of these lesions. The mild elevation in hepatic enzymes observed in laboratory tests did not indicate a specific etiology and may have been secondary to biliary tract compression caused by the mesenteric cystic mass.

Among the reported cases in the literature, Yang et al. [19] described the largest lesion, measuring 30 cm × 20 cm × 15 cm, in a 23‐year‐old male who underwent complete excision with preservation of adjacent organs. Seki et al. [20] reported a 20 cm × 15 cm × 8 cm lesion in a 54‐year‐old woman presenting with acute abdomen, which required resection of a segment of jejunum. More recently, Yin et al. [8] documented a lesion measuring 17.2 cm × 14.5 cm × 8.1 cm in a 66‐year‐old man. Hornick and Fletcher [21] reported a series of seven intra‐abdominal cystic lymphangiomas in adults, with three cases involving the small intestinal mesentery and lesion sizes ranging from 8 to 20 cm (median, 15 cm). Su et al. [22] described eight mesenteric lymphangiomas in their series of 17 adult patients, with a median tumor diameter of 12.0 cm (Table 1).

**Table 1:** Summary of previously reported adult cases of mesenteric cystic lymphangioma, including patient age, lesion size, anatomical location, and surgical approach.

Author (year)	Patient age	Lesion size	Location	Surgical approach
Yang et al. [19] (2014)	23	30 cm × 20 cm × 15 cm	Mesentery	Open (laparotomy)
Seki et al. [20] (1998)	54	20 cm × 15 cm × 8 cm	Jejunum and mesentery	Open (laparotomy)
Yin et al. [8] (2025)	66	17.2 cm × 14.5 cm × 8.1 cm	Small intestinal mesentery	Open (laparotomy)
Hornick and Fletcher [21] (2005)	Adults (median of 42)	8–20 cm (median of 15 cm)	Small intestinal mesentery (3 of 7 cases)	Not specified
Su et al. [22] (2007)	Adults (median of 39)	8–20 cm (median of 12 cm)	Mesentery (8 patients)	Open (laparotomy)

The present case, with a lesion measuring 21 cm × 20 cm × 16 cm, represents one of the largest giant mesenteric cystic lymphangiomas reported in adults. Only the case described by Yang et al. [19] exceeded these dimensions. The rarity of such lesions, combined with the scarcity of detailed reports in literature, underscores the importance of publishing well‐documented cases. Comprehensive case descriptions contribute to the collective clinical experience and may serve as valuable references for clinicians facing similar presentations, particularly regarding surgical planning, intraoperative decision‐making, and postoperative management.

CT, and especially MRI, are crucial for accurately characterizing these lesions [8, 11]. In this case, CT initially revealed a large cystic mass with significant anatomical relationships, prompting further evaluation using MRI. This examination confirmed the multiloculated cystic nature of the lesion, supporting the presumptive diagnosis of mesenteric lymphangioma. The high accuracy of MRI in characterizing abdominal cystic lesions highlights its importance as a key imaging tool for investigating abdominal masses of uncertain origin, providing valuable information for preoperative planning [7].

Laboratory tests have limited utility in establishing a diagnosis of mesenteric lymphangioma. Additional procedures, such as FNA with cytologic analysis or exploratory laparoscopy, are rarely required for cyst characterization [12, 23]. In our case, the highly specific imaging features identified on MRI provided sufficient diagnostic confidence, thereby eliminating the need for preoperative FNA. Surgical management was performed accurately and resulted in a definitive cure.

Surgical treatment was chosen because of the lesion’s considerable size and the potential for complications such as compression of adjacent organs, torsion, hemorrhage, and cyst rupture. In this case, total tumor removal was achieved without any additional visceral resection or postoperative complications. No associated retroperitoneal lymphadenectomy was performed, and only tumor‐encased lymph nodes were removed. Recent guidelines for the general management of lymphangiomas do not recommend performing an associated lymphadenectomy. Nevertheless, at the time, there was no evidence to support or contraindicate lymphadenectomy in cases of adult giant mesenteric lymphangiomas.

Although minimally invasive surgery has been increasingly reported for abdominal cystic lymphangiomas [24, 25], the role of laparoscopy in giant lesions remains controversial. In the Chinese cohort reported by Xiao et al. [26], both laparotomy and laparoscopy were feasible, but open surgery was preferentially adopted for larger and more complex cysts, with a trend toward increased operative blood loss and longer postoperative stay in the open group, albeit without statistical significance. Similarly, Makni et al. [27] described open resection as the predominant approach in their series of intra‐abdominal cystic lymphangiomas, with most patients undergoing laparotomy. Therefore, despite advances in minimally invasive techniques, open surgery remains an important therapeutic option, particularly in cases where complete resection may be technically challenging [26, 27].

Laparoscopic resection is the preferred surgical approach for mesenteric lymphangiomas when the lesion is readily accessible, demonstrates minimal adherence to vital structures, and permits complete excision without necessitating major organ resection [16, 27]. In cases of extensive local invasion or significant anatomical complexity, conversion to open laparotomy or a laparoscopic‐assisted approach may be required to ensure safe and complete tumor removal, as seen in other related cases of giant cystic mesentery lymphangiomas (Table 1).

The conventional open approach was chosen because of the considerable size of the lesion and its intimate relationship with the major mesenteric vessels, biliary tract, and adjacent organs. Although minimally invasive techniques have been described for smaller and well‐circumscribed lymphangiomas, we considered that laparoscopy might limit adequate exposure and safe manipulation in this case. Open laparotomy enabled complete en bloc resection and thorough exploration of the abdominal cavity, which also allowed the identification and excision of an additional focus of the disease.

The definitive diagnosis was confirmed through histopathological examination after surgical removal, which revealed dilated lymphatic structures consistent with cystic lymphangioma.

Immunohistochemical studies using lymphatic endothelial markers, such as factor VIII‐related antigen and D2‐40, can aid in confirming the diagnosis [28]. However, since these tests were not available at our institution at the time, the diagnosis was primarily based on histopathological findings, such as dilated lymphoid channels within the stroma of the specimen.

The differential diagnosis of mesenteric lymphangioma is extensive, making a definitive preoperative diagnosis challenging [29]. It includes nonneoplastic lesions, such as lymphoceles and pancreatic pseudocysts, and benign neoplasms, such as benign cystic mesothelioma and teratomas [29, 30]. Most importantly, malignancies, including pseudomyxoma peritonei, malignant mesothelioma, and solid tumors with cystic degeneration, must be excluded [29, 30]. The absence of a nodular or thick wall or septal enhancement is a crucial feature favoring lymphangioma over malignancy [30].

Mesenteric cystic lymphangioma in adults is a rare condition that should be considered in the differential diagnosis of abdominal cystic lesions. Imaging studies, particularly MRI, are valuable for lesion characterization and preoperative planning; however, histopathological examination remains the gold standard for a definitive diagnosis. Early recognition and complete surgical excision are essential to prevent complications and reduce the risk of recurrence, particularly for symptomatic or large lesions. Regular postoperative follow‐up is recommended to monitor potential relapse.

## Author Contributions

All authors contributed to the study’s conception and design. Material preparation, data collection, and analysis were performed by Abdo Imad El Tawil, Beatriz Arnaut Mendes, Leandro Alencar Furtado Machoski, and Eduardo José Brommelstroet Ramos. Eduardo José Brommelstroet Ramos, Micheli Fortunato Domingos, and João Carlos Chiquetto Filho were directly involved with patient care and management. The first draft of the manuscript was written by Abdo Imad El Tawil, Beatriz Arnaut Mendes, and Leandro Alencar Furtado Machoski, and all authors commented on previous versions of the manuscript.

## Funding

The authors declare that no funds, grants, or other support were received during the preparation of this manuscript.

## Disclosure

All the authors have read and approved the final manuscript.

## Consent

Informed consent was obtained from the patient included in this study. The authors affirm that this is a single case report and does not involve human research. The patient provided informed consent for the publication of this work.

## Conflicts of Interest

The authors declare no conflicts of interest.

## Data Availability

The data supporting the findings of this study are available upon request from the corresponding author. The data are not publicly available because of privacy or ethical restrictions.

## References

[1] Liew S. C. C. , Glenn D. C. , and Storey D. W. , Mesenteric Cyst, Australian and New Zealand Journal of Surgery. (1994) 64, no. 11, 741–744, 10.1111/j.1445-2197.1994.tb04530.x, 2-s2.0-0028007209. 7945079

[2] Chung M. A. , Brandt M. L. , St-Vil D. , and Yazbeck S. , Mesenteric Cysts in Children, Journal of Pediatric Surgery. (1991) 26, no. 11, 1306–1308, 10.1016/0022-3468(91)90606-T, 2-s2.0-0026328759. 1812263

[3] de Cardoso Filho F. A. , Landim F. M. , and Perdigão F. B. , Linfangioma cístico do mesentério: uma rara apresentação de abdômen agudo, Revista do Colégio Brasileiro de Cirurgiões. (2000) 27, no. 2, 136–137, 10.1590/S0100-69912000000200014.

[4] Saxena A. , Kakodkar P. , and Zhang D. , et al.Intra-Abdominal Multi Cystic Lymphangiomas: A Case Series With Adult and Pediatric Literature Review, Archives of Clinical Gastroenterology. (2024) 10, no. 3, 027–039, 10.17352/2455-2283.000125.

[5] Gunadi N. , Kashogi G. , Prasetya D. , Fauzi A. R. , Daryanto E. , and Dwihantoro A. , Pediatric Patients With Mesenteric Cystic Lymphangioma: A Case Series, International Journal of Surgery Case Reports. (2019) 64, no. 3, 89–93, 10.1016/j.ijscr.2019.09.034, 2-s2.0-85073114919. 31622933 PMC6796738

[6] Konen O. , Rathaus V. , and Dlugy E. , et al.Childhood Abdominal Cystic Lymphangioma, Pediatric Radiology. (2002) 32, no. 2, 88–94, 10.1007/s00247-001-0612-4, 2-s2.0-0036935391. 11819071

[7] Thiam O. , Faye P. M. , and Niasse A. , et al.Cystic Mesenteric Lymphangioma: A Case Report, International Journal of Surgery Case Reports. (2019) 61, 318–321, 10.1016/j.ijscr.2019.07.051, 2-s2.0-85070850066. 31399398 PMC6718053

[8] Yin W. , Yu R. , and Xia D. , A Gaint Mesenteric Cystic Lymphangioma of Small Intestinal in an Adult: A Case Report and Literature Review, Medicine. (2025) 104, no. 21, 10.1097/MD.0000000000042394, e42394. 40419877 PMC12113915

[9] Miceli A. and Stewart K. M. , Lymphangioma, 2023, StatPearls Publishing. 29261940

[10] Abdulraheem A. K. , Al Sharie A. H. , Al Shalakhti M. H. , Alayoub S. Y. , Al-Domaidat H. M. , and El-Qawasmeh A. E. , Mesenteric Cystic Lymphangioma: A Case Report, International Journal of Surgery Case Reports. (2021) 80, no. 7, 10.1016/j.ijscr.2021.105659, 105659. 33636409 PMC7918257

[11] Mabrut J. Y. , Grandjean J. P. , and Henry L. , et al.Les Lymphangiomes Kystiques du mésentère et du méso-côlon. Prise en Charge Diagnostique et thérapeutique, Annales De Chirurgie. (2002) 127, no. 5, 343–349, 10.1016/S0003-3944(02)00770-8, 2-s2.0-0036273193. 12094416

[12] Prior-Rosas J. E. , Mejía-Ruíz B. , Magdaleno-Becerra B. A. , Nava-Tenorio C. G. , Alonso-Domínguez S. M. , and Botello-Ortiz G. E. , Giant Benign Mesenteric Cysts (Mesothelioma and Lymphangioma): A Report of Two Cases, International Journal of Surgery Case Reports. (2024) 125, 10.1016/j.ijscr.2024.110587, 110587. 39549585 PMC11614839

[13] Stewart C. J. R. , Chan T. , and Platten M. , Acquired Lymphangiectasia (’Lymphangioma Circumscriptum’) of the Vulva: A Report of Eight Cases, Pathology. (2009) 41, no. 5, 448–453, 10.1080/00313020902885052, 2-s2.0-70350633015. 19396719

[14] Thiam O. , Faye P. M. , Sarr I. S. , Niasse A. , and Dieng M. , Splenic Lymphangioma, Pan African Medical Journal. (2019) 33, 132.31558931

[15] Yang D. M. , Jung D. H. , and Kim H. , et al.Retroperitoneal Cystic Masses: CT, Clinical, and Pathological Findings and Literature Review, RadioGraphics. (2004) 24, no. 5, 1353–1365, 10.1148/rg.245045017, 2-s2.0-4944265241. 15371613

[16] Nagano H. , Kimura T. , Iida A. , Togawa T. , Goi T. , and Sato Y. , Cystic Lymphangioma in the Peripheral Jejunal Mesentery in an Adult and Excision With Laparoscopic-Assisted Surgery: A Case Report, World Journal of Surgical Oncology. (2019) 24, no. 1. 10.1186/s12957-019-1713-6 PMC6814111 31651341

[17] Banerjee J. K. , Bharathi R. S. , Venkatesan S. , and Singh G. , Abdominal Lymphangioma, Medical Journal Armed Forces India. (2016) 72, no. 1, S70–S73, 10.1016/j.mjafi.2016.03.004, 2-s2.0-85007238034. 28050075 PMC5192212

[18] Wani I. , Mesenteric Lymphangioma in Adult: A Case Series With a Review of the Literature, Digestive Diseases and Sciences. (2009) 54, no. 12, 2758–2762, 10.1007/s10620-008-0674-3, 2-s2.0-70450260880. 19142726

[19] Yang Y. , Cai Y. , Li Z. , Fang Y. , Xiang J. , and Chen Z. , Mesenteric Lymphatic Hygroma in Adults: A Case Report With a Review of the Literature, Oncology Letters. (2014) 7, no. 3, 709–712, 10.3892/ol.2013.1778, 2-s2.0-84892968381. 24527076 PMC3919917

[20] Seki H. , Ueda T. , Kasuya T. , Kotanagi H. , and Tamura T. , Lymphangioma of the Jejunum and Mesentery Presenting With Acute Abdomen in an Adult, Journal of Gastroenterology. (1998) 33, no. 1, 107–111, 10.1007/s005350050053, 2-s2.0-0031914570. 9497231

[21] Hornick J. L. and Fletcher C. D. , Intraabdominal Cystic Lymphangiomas Obscured by Marked Superimposed Reactive Changes: Clinicopathological Analysis of a Series, Human Pathology. (2005) 36, no. 4, 426–432, 10.1016/j.humpath.2005.02.007, 2-s2.0-17844410647. 15892005

[22] Su C. M. , Yu M. C. , Chen H. Y. , Tseng J. H. , Jan Y. Y. , and Chen M. F. , Single-Centre Results of Treatment of Retroperitoneal and Mesenteric Cystic Lymphangiomas, Digestive Surgery. (2007) 24, no. 3, 181–185, 10.1159/000102896, 2-s2.0-34347212867. 17522464

[23] Miljković D. , Gmijović D. , Radojković M. , Gligorijević J. , and Radovanović Z. , Mesenteric Cyst, Archive of Oncology. (2007) 15, no. 3-4, 91–93, 10.2298/AOO0704091M, 2-s2.0-50049135328.

[24] Târcoveanu E. , Moldovanu R. , Bradea C. , Vlad N. , Ciobanu D. , and Vasilescu A. , Laparoscopic Treatment of Intraabdominal Cystic Lymphangioma, Chirurgia. (2016) 111, no. 3, 236–241. 27452935

[25] Sato T. , Matsuo Y. , and Shiga K. , et al.Laparoscopic Resection of Retroperitoneal Lymphangioma Around the Pancreas: A Case Report and Review of the Literature, Journal of Medical Case Reports. (2015) 9, no. 1, 10.1186/s13256-015-0760-z, 2-s2.0-84949544085, 279. 26651336 PMC4675055

[26] Xiao J. , Shao Y. , Zhu S. , and He X. , Characteristics of Adult Abdominal Cystic Lymphangioma: A Single-Center Chinese Cohort of 12 Cases, BMC Gastroenterology. (2020) 20, no. 1, 10.1186/s12876-020-01388-8, 244. 32727377 PMC7391610

[27] Makni A. , Chebbi F. , and Fetirich F. , et al.Surgical Management of Intra-Abdominal Cystic Lymphangioma. Report of 20 Cases, World Journal of Surgery. (2012) 36, no. 5, 1037–1043, 10.1007/s00268-012-1515-2, 2-s2.0-84863717966. 22358782

[28] Thapa S. , Sharma A. , and Upreti D. , et al.A Huge Mesenteric Lymphangioma Presenting as a Small Bowel Volvulus in a Paediatric Patient: A Case Report, Case Reports in Pathology. (2022) 2022, no. 2, 10.1155/2022/3033705, 3033705. 35620582 PMC9130006

[29] Marco M. D. , Grassi E. , Vecchiarelli S. , Durante S. , Macchini M. , and Biasco G. , Retroperitoneal Lymphangioma: A Report of 2 Cases and a Review of the Literature Regarding the Differential Diagnoses of Retroperitoneal Cystic Masses, Oncology Letters. (2016) 22, no. 5, 3161–3166. 10.3892/ol.2016.4367 PMC4841079 27123082

[30] Raufaste Tistet M. , Ernst O. , Lanchou M. , Vermersch M. , and Lebert P. , Imaging Features, Complications and Differential Diagnoses of Abdominal Cystic Lymphangiomas, Abdominal Radiology. (2020) 45, no. 11, 3589–3607, 10.1007/s00261-020-02525-3. 32296900

